# How systemic cognition enables epistemic engineering

**DOI:** 10.3389/frai.2022.960384

**Published:** 2023-02-07

**Authors:** Stephen J. Cowley, Rasmus Gahrn-Andersen

**Affiliations:** Department of Language and Communication, University of Southern Denmark, Slagelse, Denmark

**Keywords:** distributed cognition, social organizing, simplexity, systemic cognition, radical embodied cognitive science, pre-reflective experience, vicariance, evoneering

## Abstract

Epistemic engineering arises as systems and their parts develop functionality that is construed as valid knowledge. By hypothesis, epistemic engineering is a basic evolutionary principle. It ensures that not only living systems identify the differences that make differences but also ensure that distributed control enables them to *construct* epistemic change. In tracking such outcomes in human life, we stress that humans act within poly-centered, distributed systems. Similar to how people can act as inert parts of a system, they also actively bring forth intents and vicariant effects. Human cognitive agents use the systemic function to construct epistemic novelties. In the illustration, we used a published experimental study of a cyborg cockroach to consider how an evoneered system enables a human subject to perform as an adaptor with some “thought control” over the animal. Within a wide system, brains enable the techniques to arise *ex novo* as they attune to the dictates of a device. Human parts act as adaptors that simplify the task. In scaling up, we turn to a case of organizational cognition. We track how adaptor functions spread when drone-based data are brought to the maintenance department of a Danish utility company. While pivoting on how system operators combine experience with the use of software, their expertise sets off epistemically engineered results across the company and beyond. Vicariant effects emerge under the poly-centered control of brains, persons, equipment, and institutional wholes. As a part of culture, epistemic engineering works by reducing entropy.

## 1. Introduction

In Europe and America, knowing is often ascribed to an organism, body, mind, or brain. In contrast to, say, Chinese or African traditions, the individual is treated as the locus of both know-how and reason. In making a link between anthropology and computational models, Hutchins (1996) brings new light to how collective knowing enables to inform human agency. In allowing cognitive distribution, he traces epistemic outcomes across systems that lack a single locus of control. When rowing canoes across the Pacific or, indeed, bringing a ship into port, people link up beliefs, devices, observations, and acting within culturally distributed systems. Knowing includes—but is not generated by—individual actors. In applying the view to science, Giere (2004) invokes how the Hubble spacecraft enabled distributed systems to bring forth new knowledge of the universe. Like other organized knowledge, poly-centered systems enable science to arise through what Giere (2004) calls “human cognitive agents.” In what follows, we radicalize such views by tracking how wide systems can affect the epistemic agency of living human beings.

Primate intelligence is predominantly social (Jolly, 1966; Humphrey, 1976) and, in the last million years or so, hominins and eco-systems have co-evolved (Sterelny, 2007). Bodies and, especially, brains have brought humans the extreme plasticity that sustains practices such as trade (Ross, 2012). In Hutchins's (1996) terms, practices inform the distributed cognitive systems that link artifacts, language, and ways of acting. Hence, they include what Malafouris (2013, 2019) calls *material engagement*: in using materials such as clay, we draw upon cultural resources such as norms and conventions as bodily promptings enable us to use techniques, skills, and methods. For Malafouris, “enactive signification” arises as parameters co-function to nudge a person to substitute one way of acting with another. Humans gain flexibility and construct epistemic powers as they actualize social practices. They perform roles and develop styles that create diversity that uses a trick of *vicariance* or how one can “perform the same tasks with different systems, solutions or behaviors” (Berthoz and Tramus, 2015, p. 1–2). Crucially, since vicariance serves bodies, brains, and social activities (Cowley and Gahrn-Andersen, 2022), it creates novelty by reducing entropy or uncertainty (usually, if not always, by changing the parameters of a system). Vicariant effects spread across bodily modalities, social groups, and neural organization and, as a result, parties gain as epistemic change self-fabricates within cognitive systems.

In pursuing how such vicariant effects are brought about, the article begins with a “minimal” case. We describe how, in an experimental setting, a system sets off epistemic change as a person comes to exert “thought control” over a cockroach. Agency links an engineered system, human-cockroach interdependencies, pre-reflective experience, and a brain that constructs and sustains bodily techniques. Highlighting the systemic, we emphasize how the human adaptor uses cognition beyond the body. Later, we compare the neural parameter setting of the cockroach experiment to how vicariant effects spread when drones were introduced to a Danish utility company. In both cases, people reduce entropy (uncertainty) within wide cognitive systems as, often without knowing why, they set off effects that serve a wider system: vicariant outcomes thus transform both individual performance and the company task regime.

## 2. Cognition—The role of “knowledge” for systems

A distributed perspective on cognition (Hutchins, 1996; Rogers, 1997; Perry, 2013) first emerged as a counterpoint to core tenets of orthodox cognitivism (e.g., Fodor, 1975; Marr, 1982; Searle, 1992). It does so in that the classic cognitive view treats the organism as the “source” of intelligent behavior. In philosophical guise, knowledge is ascribed to sense impressions, mind, and reason; by contrast, with cognitive science, attention falls on learning, computation, sense-making, organism-environment coupling, etc. Turning to working environments, Hutchins showed that, in many cases, such models are demonstrably inadequate. There is no organismic source of cognition in, say, navigation. Rather, people incontrovertibly draw on cultural resources and wide systems (Wilson, 2004) to achieve epistemic outcomes. Socially organized activity is a dynamical interplay of agents and environments which link cognitive practices with, above all technologies and external representation media. In a distributed system, social practices or organizations sustain heterogeneous kinds of processes. The distributed perspective thus applies to practices as diverse as, say, crime scene investigation (Baber, 2010), medical situation awareness (Fioratou et al., 2016), insight problem-solving (Vallée-Tourangeau and Wrightman, 2010), or, indeed, how a daughter decorously tries to quieten her mother (Cowley, 2014).

The entire cognitive system unites a myriad of parts as “inner and external” resources co-function in diverse ways (cf. Michaelian and Sutton, 2013, p. 10). As Hutchins (2014) came to phrase it in theoretically oriented work, the perspective applies to all of human cognition: it characterizes “the microprocesses of interaction across the diverse components of these distributed and heterogeneous cognitive systems” (Hutchins, 2014, p. 5). Yet, as Hutchins notes, his own early work views “cognitive processes in terms of the propagation and transformation of representations” (Hutchins, 2001, p. 2068). Hence, proponents of the distributed perspective who retain a traditional model of representations find themselves committed to the “source” view of orthodox cognitivism (for a criticism, see Hutto et al., 2014). Placing intent in the brain, they treat cognizers as parties that propagate and transform “particular representational states across distinct (internal and external) media” (Michaelian and Sutton, 2013, p. 5). Whereas, Hutchins began with a focus on representations in a literal sense (Hutchins, 1996, p. 363–364), he later shifts to a more liberal view. Hence, far from addressing the role of living agency in cognition – or how intent arises – later work (Hutchins, 2020) still focuses on how externalized resources extend how people act as they perform social roles and rely on interactions. He explicitly suggests that “distributed cognition is not a kind of cognition at all, it is a perspective on cognition.” His concern is with, not explaining cognition or the role of bodies in epistemic change, but, rather, how “participants to an interaction coinhabit a shared environment” (2020, p. 375). Very plausibly, Hutchins adopts the view that “interaction is the basis for the distribution of cognitive labor” (2020, p. 377). As an ethnographer, albeit an unorthodox one, he approaches people as social actors. Leaving aside issues of intent, he can overlook *how* agency changes and, on methodological grounds, changes in cultural operations. Since he asks how participants contribute to procedures, he reduces language and agency to their role in task performance. Others are more concerned with individual responsibility (Jones, 2013) or how looser systems depend on language, knowledge, and expertise (Perry, 2013). In seeking to deal with the tension, Baber et al. (2014), for example, use the concept of “affordances” to allow individual control of tools within a “person–environment–tool–object system” (p. 10). Adopting Turvey's (1992) view of affordance (Gibson, 1979), Baber et al. allow for individual expertise in control:

Even if there are regions that are active under specific conditions, the skill of the expert tool user comes from the ability to control their activity with sufficient spare capacity to cope with future demands and to respond to the changing context in which they are using the tools to effect changes in the object being worked on. (Baber et al., 2014, p. 12)

In making individual skills and expertise partly constitutive of distributed processes, Baber et al. identified the collective-individual tension that runs through research on distributed cognition. The focus on outcomes can lead one to highlight, not individual doings, but a collective effort. For instance, Hutchins reports on how the crew of the USS Palau dealt with the issues relating to the loss of main steam (Hutchins, 1996). He traces the outcomes to how tightly coupled practices are structured around the well-understood/defined task of managing how the vessel is brought to anchor. Hutchins writes:

The safe arrival of the Palau at anchor was due in large part to the exceptional seamanship of the bridge crew, especially the navigator. But no single individual on the bridge acting alone–neither the captain nor the navigator nor the quartermaster chief supervising the navigation team–could have kept control of the ship and brought it safely to anchor. (p. 5)

Although Hutchins (1996) recognizes the seamanship of the navigator, his ethnography of the supra-entity highlights interaction and participant roles. Hence, Hutchins plays down individuals, intents and propensities, how skills arise, or how they are selected. This is because, in a task context, the right choices are simply assumed. Furthermore, it is by treating a person as a social actor (not a source of cognizing) that the distributed perspective breaks with classic views. Later, we show how it allows emphasis on autonomy to be replaced by a view of agency as using poly-centered and diachronic control. Indeed, even on a standard view, this is implied where a system:

dynamically reconfigures itself to bring subsystems into functional coordination. Many of the subsystems lie outside individual minds; in distributed cognition, interactions between people as they work with external resources are as important as the processes of individual cognition (Lintern, 2007, p. 398).

Control arises as the system co-configures its functions such that tasks are successfully accomplished. Classically, it uses extant equipment, routines, procedures, etc. or, as for Latour (2007), human and non-human parts to serve as actors (“actants”). In what follows, unlike Latour and Hutchins, we will turn to how living human bodies function as parts of wide systems.

Starting with social actors allows a single “level of analysis” to apply to organizations, practices, and ways of acting. Turning from control, Hutchins (1996) identifies distributed cognition with tightly coupled practices that, in later work (Hutchins, 2014, 2020), are explicitly said to ground all of human cognition. He uses what Cheon (2014) calls a “task-specification requirement” where activity is “distributed” around a clearly specified and collectively understood task. Such a view is exemplified by the malfunction in the steam whistle where, for the crew, their task becomes that of finding a functional substitute or vicariant solution to warning an approaching sailboat of possible collision (Hutchins, 1996, p. 4). As in Marr's (1982) work on vision, a cognitive task is computationally defined and, given formal description, separated from a (presumed) implementational level. Even Hutchins (2014) retains this view in recent work on the details of cockpit control: here too, he leaves aside implementation to focus on actions: thus, in Weibel et al. (2012), the use of eye-tracking data is reported. However, it serves to pursue, for example, the meaning of the pilot's “light touching of the front edge of left thrust lever with the side of the pinky finger on his right hand, bumping it lightly in the direction of reduced thrust” (p. 112). For methodological reasons, as Gahrn-Andersen (2021) shows, the object of study concerns how humans act as parts of well-defined cognitive systems. In other words, given an extant epistemic definition of the task, the whole system (e.g., practice, organization) is viewed as a stable, supervening entity. Control draws on predictable functionality to ensure that what is described *counts as valid knowledge*. Yet, a high price is paid by starting with a systemic whole. Human individuals become social operators in unchanging systems. Thus, for Afeltowicz and Wachowski (2015), the approach fails to qualify as a cognitive theory because it cannot clarify how intent arises. Of course, the perspective has no such goal. However, recognition of the flaw points to the interdependency of living and non-living systems. This is prefigured by Giere (2004) who, taking the distributed perspective to science, carefully distinguishes the human cognitive agent from the whole system. Without this move, one risks assuming, with Michaelian and Sutton (2013) that “expertise is not a property of individual agents, but is built in to the constraints of the system” (Michaelian and Sutton, 2013, p. 5). Not only does one leave aside how intent emerges but also one replaces a whole system's pre-established structures and loci of control (e.g., routines) with attention to operational shifts, systemic change, expertise and the entangled, and highly variable workings of living human bodies. While their functions indeed reach beyond the sum of its parts determining proper actions, only attention to a “person-in-the-system” (Fester-Seeger, 2021) can open up how systems generate intent or use vicariant effects to achieve epistemic change.

Hutchins (2014) applies his perspective to all of human cognition by comparison to the theory of extended mind. Hence, task-based human cognition falls within the constraints of “cultural eco-systems.” He views how agents perform –act, draw, and speak –as “participants” in wider systems: hence as in earlier work, his focus is collective. Indeed, an ecosystemic focus abstracts away from actual doings and organized action. Hutchins seek to “shift the focus from ecological assemblies surrounding an individual person to cultural ecosystems operating at larger spatial and temporal scales” (2014, p. 35). Of course, at a descriptive level, he recognizes that individual participant matters (e.g., as in the case of a flight crew's visual attention which is structurally determined by the practice of preparing for descent 2014, p. 44). Theoretically, however, he emphasizes systemic stability or how existing practices are sustained. In his terms, “the stability, resilience, or persistence of a practice depends on the network of relations to other practices within which it is embedded” (p. 46). Indeed, Hutchins emphasizes a “web of cultural regularities” and, with these, the cultural practices, which sustain them (2014, p. 47). As he notes, the perspective allows practices to reduce contingencies to the extent that those familiar with a relevant ecosystem will experience similar phenomena as belonging to the same type (e.g., perceiving a line of people as a queue). Importantly, he notes how “cultural practices decrease entropy and increase the predictability of experience” (2014, p. 46). In this context, even individual learning is structurally determined by ecosystemic regularities. The perspective thus treats both individual and collective experiences as intrinsic to the operations that guarantee systemic reproduction. By implication, parts (e.g., workers or equipment) and procedures are functionally replaceable. This takes us back to our criticism of Hutchins (1996): By taking the supra-entity as given-in-advance, he fails to interrogate how epistemic shifts occur. Rather, his system is functionally indifferent to the substitution of its elements and actual ways of performance. Instead of exploring intents, systemic adjustment, change, and development, vicariance is separated from persons and systemic dysfunction or, indeed, significant operational change.

While a truism that human agency and power are socially distributed, we turn to how parameters operate as events arise in epistemic domains. Building on viewing language as distributed by how embodiment informs agency (Blair and Cowley, 2003; Cowley, 2011, 2014), we highlight systemic interdependency. Similar to what Giere (2004) shows for science or Vallée-Tourangeau and Wrightman (2010) for individual differences in mental arithmetic, we stress that persons are interdependent with non-living parts of wider systems. As illustrated below, these prompt epistemic change in, at times, neural organization and, at others, an organized task regime. A wider system induces vicariant effects as persons engage with things and each other. Each person-in-the system is a social actor (i.e., a living being and a participant) who contributes to cascading systemic change (in various scales). Often, epistemic change is triggered as an agent draws on what appears as an *ex novo* event. Turning to functional coordination and stability, we stress how distributed agency (refer to Enfield, 2013) drives epistemic change. Since this has a biosocial basis, human cognition links distributed systems to living bodies, language-activity (or languaging) and semiotic assemblages (Pennycook, 2017). In order to clarify how vicariant effects arise, we bring systemic ethnography to how, in actual cases, practices unfold. We unleash the power of tracing living human agency to how bodies (and brains) contribute as parts of wide systems. Individual agents draw on their embedding in larger wholes to shape traits a person's competencies (in the system). Hence, distributed parts enable organic and organized parameter setting as systemic function draws on what we call *epistemic engineering*. As a result, the process enables humans to use ecosocial resources in a life history of epistemic change. Coming to know this implicates routine performance that unites separable systems, various control centers (e.g., brains and computers) and modes of action.

As will be explained in section 5, our account turns from a computational (or supra-entity) level by treating human cognition as systemic and poly-centric. Accordingly, we play down pre-determined cognitive tasks and views that ascribe cognition to a single implementational source (i.e., a strictly autonomous system). Before turning to our systemic frame (Cowley and Vallée-Tourangeau, 2013, 2017; Secchi and Cowley, 2021; Secchi et al., 2023), we present two case studies of epistemic engineering. These illustrate (a) how cognitive systems require changing the loci of control and (b) how agents, in their capacity as such, draw on vicariant effects to affect the outcome of distributed systems.

## 3. The minimal case

A principle of neural re-use (Anderson, 2010) permits brains to use a body's life history as they construct bodies that develop as effective performers and, indeed, participants in distributed systems. Hence, we begin with how neural flexibility enables a person to adapt to what we call a *minimal engineered system*. Similar vicariant effects occur with, say, sensory substitution (Froese and Ortiz-Garin, 2020) or “thought” control of a prothesis (e.g., Edelman et al., 2019). While evoneered technology is often studied as of value in itself, less weight has hitherto been placed on the biotech interface or how a living brain adapts to a device. In that the results demand learned adaptation, we extend work published elsewhere (Gahrn-Andersen and Prinz, 2021) to highlight *natural* evoneering.

**Figure 1:**
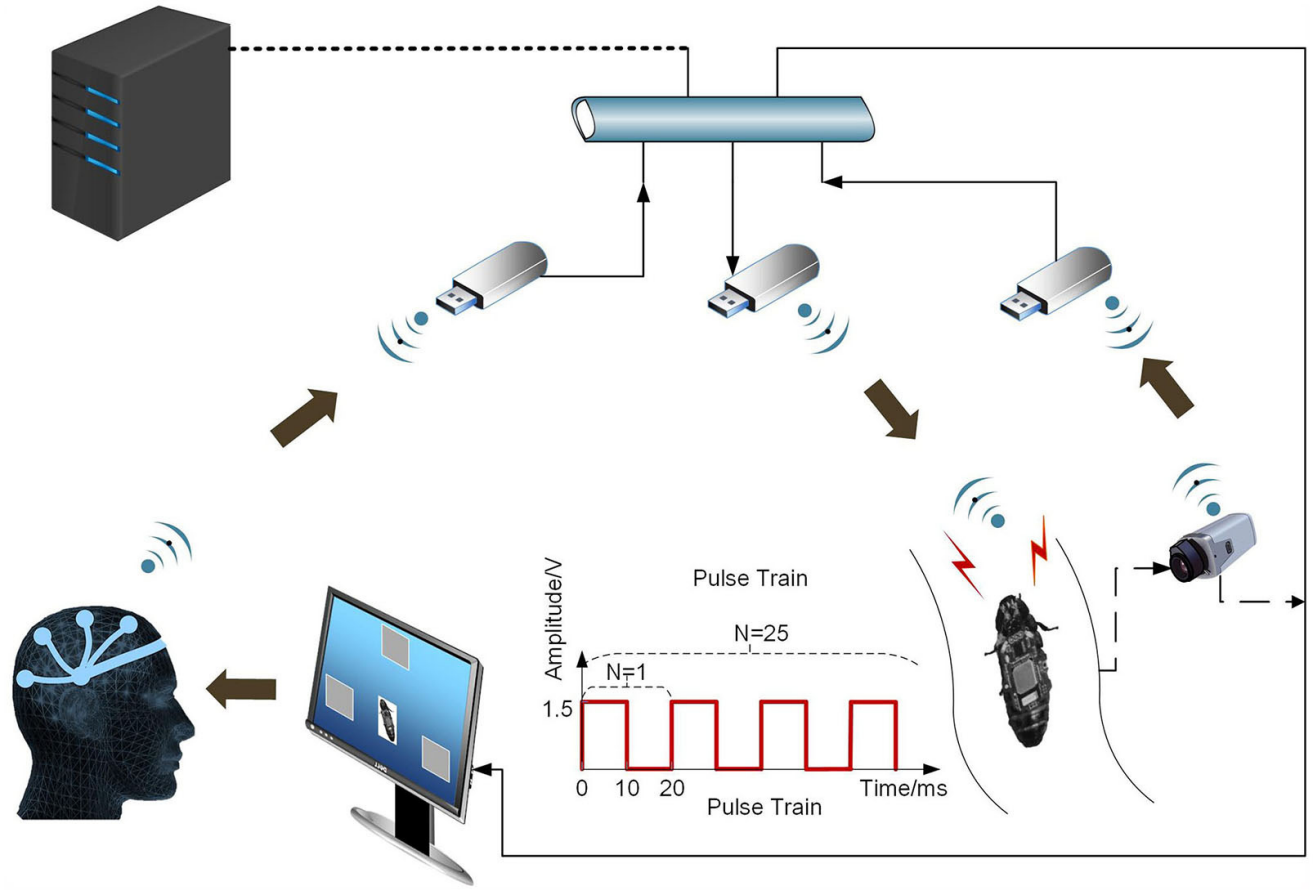
The experimental system encompasses a person, a cockroach and a steady-state visual evoked potential (SSVEP) based brain-computer interface (BCI). The distributed system works as the subject sends real time BCI commands to the cockroach as a person responds to a flickering image of the cockroach. Link to original source: https://journals.plos.org/plosone/article?id=10.1371/journal.pone.0150667.

**Figure 2:**
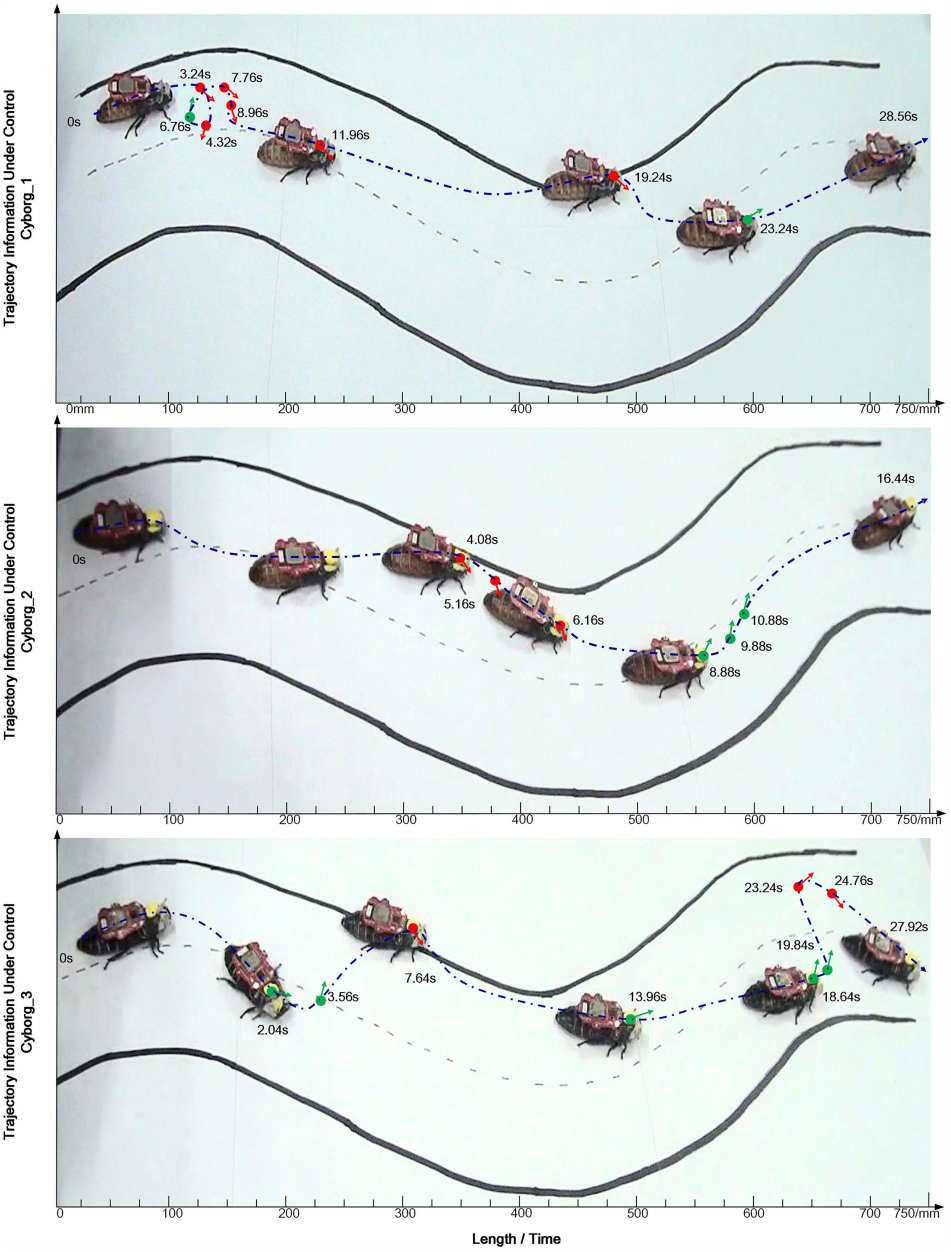
The trajectory of a cockroach moving on the S curve showing time taken. A green dot indicates a left-turn command; a red dot indicates a right-turn command. Link to original source: https://journals.plos.org/plosone/article?id=10.1371/journal.pone.0150667.

In the case of the cyborg cockroach, “thoughts” come to influence an insect's movements (Li and Zhang, 2016). Of course, this is not literally a matter of “thinking”: rather, without knowing what he or she is doing, a person manages input to the visual cortex that is monitored by an EEG device. Since this transmits to the cockroach's antenna nerve, it sets off vicariant effects. Since a result, the cockroach comes to resemble a cyborg in that it moves, to an extent, under human control. The person gains a new way of acting: he or she uses an engineered interface within a poly-centered system. As a person-adaptor controls EEG response to a moving cockroach on a flickering screen, the subject wills “thoughts” or, more precisely, generates micro-electronic input. The subject learns to will left and right movements by influencing the cockroach's antennae nerves. Building on work which showed that cockroach moves can be shaped by radio transmission of joystick manipulation (Latif and Bozkurt, 2012), Li and Zhang (2016) added the brain-to-brain interface between EEG-output and antennae nerves. In what follows, we report on an experimental study that involved three subjects and three cockroaches. This vicariant enabling device allowed subjects to learn to use “watching and willing” to nudge a moving cockroach on an S-shaped track (refer to Figure 2). In Figure 1, we present an engineering view of the poly-centered system.

While acting as a supra-system, experimenters merely offer instructions and minimal training. Though part of the whole system, they have no active role in “looking-and-willing” or thought control. Thus, in the terms of Lintern (2007), one can ask how the whole “dynamically reconfigures itself” (p. 398). In so doing, we focus on how epistemic change arises as a subject gains some control over the cockroach. In such a case, systems and parts enable vicariant effects as a subject masters what we call a *technique*. In this “minimal” epistemic engineering, the subject (and the brain) connect: (a) how a person *assesses*/*manages* watching-and-willing and, thus, the adaptor's EEG output^1^ and (b) how input to the antennas' nerves affects cockroach movements. If successful, the poly-centered system achieves “functional coordination” between looking, neural activity, the engineered adaptor, and the cockroach. In Lintern's (2007) terms, “external resources are as important as the processes of individual cognition” (ibid).

In producing EEG output *for* the cockroach, a human subject assesses cockroach moves while willing changes in cockroach movements (refer to Figure 2). Hence, adaptors and “thoughts” (or EEG measures) come to anticipate cockroach activity. Given repetition and experience, the human gains techniques: in an enlanguaged world, participants grasp the following: (1) what the task is; and (2) what has to be done. However, since one cannot know (in advance) what it is like to move a cyborg cockroach, techniques can only arise *ex novo*. Even if much depends on what we call skills (and can be described by theories like predictive processing), the vicariant effects do not *reduce* to brain side process. It is only as part of a brain-in-a-wide (or poly-centered) system that an engineered system can use a “composite device” constituted by the setting (and, ultimately, the work of the experimenters). In time, the accomplished use of the device and cockroach brings “synergism and functionality” to the person (Gahrn-Andersen and Prinz, 2021) who performs the experiment. Far from reducing to learning, one gains epistemic power (know-how) that is entirely dependent on the whole system: one draws on interdependencies (and repetition) in coming to act with a new kind of intent.

As Li and Zhang note, the adaptor shows “stable and continuous high levels of accuracy in both ‘sender' and ‘receiver' sides” (2016, p. 15)^2^. Accordingly, to address the rise of synergies and functionality, we focused on, first, the measures of cockroach sensitivity to micro-electronic prompts (cyborg response accuracy) and, second, human success in keeping the insect within boundaries (human success rate). Table 1 presents selected findings from those reported in detail in the original paper.

**Table 1:** Success in controlling cockroach moves.

	**Cyborg response accuracy (%)**	**Human success rate (%)**
Cockroach 1	93.1	33.3
Cockroach 2	82.4	20.0
Cockroach 3	82.9	6.7

Although one cockroach reduces the human success rate, broadly, human “thought” sets off high cyborg response accuracy. Tongue in cheek, the authors mention cockroach three's “self-willingness” or, strictly, the role of extraneous variables. Crucially, given the human success of about 20%, the task is not easy. Given this fact^3^, we treat variability as showing, first, the scope for learning and, second, marked individual differences. It is striking that human subject three has the most accurate EEG classification, the best cyborg responding, the highest success rate, and alone, some success with cockroach three. We infer that much depends on managing how the adaptor bridges between a human brain and the cockroach's antennae nerves (i.e., human-centered control of EEG input). In spite of cyborg tendencies, the cockroach is no automaton. In contrast, humans must learn to use the adaptor in task-specific ways. Since these require both motivation for success and a grasp of the problem (but *not* what to do), the techniques involve more than learning. Rather, one must ask how an adaptor shapes vicariant effects in a novel task.

**Figure 3:**
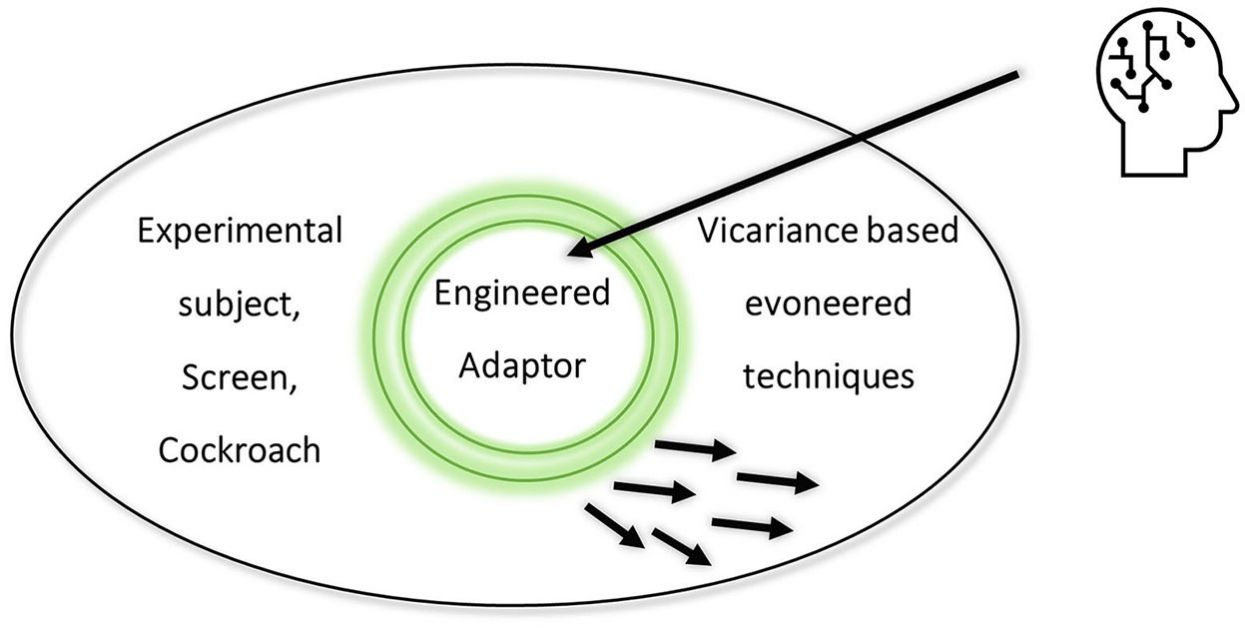
The experimenter (human head) designs a system with an engineered adaptor. As a whole adaptor system, the person, screen, and cockroach co-constitutively draw on natural evoneering.

Even if training improves skills, techniques develop and, as the success rate shows, no knack emerges. While the device sends “instructions” to the cockroach (given high response accuracy), human “thought” is subtle. Far from being a means to an end or a functional tool, the engineered system empowers the subject as an adaptor. It brings the once impossible within reach as perceptual assessment becomes part of willing a cockroach to move. Given the device, a brain-in-the-system synthesizes the *ways* of adapting (see Figure 3). As in the classic work on Tetris, the engineered system prompts the self-fabrication of epistemic powers (Kirsh and Maglio, 1994). In spite of the device's novelty, the resulting techniques use “tacit and overt controlling capacities” that allow “purposeful pre-reflective (bio)mechanical execution” (Gahrn-Andersen and Prinz, 2021). Importantly, “willing a move” must *feel* like something (for the person-in-the-system). Hence, the pre-reflective can contribute to epistemic effects as a person with a brain-operating-in-a-wide system sets off tacit neuronal tinkering. In the terms of Gahrn-Andersen and Prinz (2021), the device affects a “state of being” through “subconscious adaptation and fine tuning of neuronal circuits” (p. 110). In short, reuse enables the brain to self-design techniques for human control of the cockroach^4^.

While based on trial and error, the technique is not reducible to the “law of effect” (Dennett, 1975). Rather, as epistemic engineering, the brain gains functionality that acts as valid knowledge that is oriented to, not just a stimulus, but also the adaptor-person. Within the poly-centered system, the results attune the brain-in-the system to watching and willing. The cockroach “part” enables reinforcement to calibrate how a phenotype is extended by a system that couples an engineered adaptor, neural activity, and the pre-reflective. Hence, this constitutes natural evoneering. In the terms of Dennett's (2017) heuristics, the person needs more complexity than a Skinnerian agent but not the “inner environment” of its Popperian counterpart^5^. Rather, the brain reuses old tricks that link distributed agency with vicariance. Persons use wide systems such that, without knowing what they are doing, they bring purposefulness to learning. In Dennettian vein, one might call them Tolman agents who act with intent (i.e., *as if* they were purposeful)^6^. Just as in acting as a Morse operator (Cowley, 2019), the pre-reflective shapes techniques in a person part of a wide system. As in Tetris (Kirsh and Maglio, 1994), persons-cum-brains use the feel of *attending to the perceived*. Techniques use recursive trial and error to connect cognitive events with the feeling of what happens (Damasio, 1999). As a result of actualizing practices, experimental subjects draw on brains to self-fabricate techniques that allow for reasonable task performance.

When the engineer adds vicariant systems (e.g., a screen and EEG device) to human-cockroach engagement, the human part of the system can direct “input” to the adaptor (refer to Figure 1). What is possible is transformed: natural evoneering enables a novel technique.

Over time, the subject's brain gives rise to techniques based on seeing how the cockroach moves. Far from reducing to stimulus-response or planned action, a living human subject uses “thoughts” as attending to how the seen sets off retrojecting. The anticipative results trigger learned parameters and EEG measures, which act as output *for* a cockroach. With training and experience, humans alter how the agency is distributed between the body, devices, and the cockroach. The human uses the pre-reflective – or: the conscious but not reflectively conscious – in the entirely innovative engagement with an engineered device. Given familiarity with a cockroach-in-the-system, the pre-reflective sets off prompts and thus vicariant effects. Cognizing is evoneered across a brain that attunes to a screen and EEG device as the person-adaptor gains know-how. As a result, pre-reflective experience triggers neurophysiological events or, loosely, “thoughts.” In such a case, we meet the challenge set by Afeltowicz and Wachowski (2015): the emergence of intent (or the purposeful actions of the human) uses the interdependencies of a motivated poly-centric system. Novel behavior draws on a history that links the pre-reflective, neural activity, use of an adaptor, and contingent effects. The system's world-side resources (the adaptor-and-cyborg cockroach) use brain-side systems to shape the feeling of what happens to grant human subjects techniques. Hence, the case of minimal epistemic engineering relies on actualizing a social practice whose functionality appears to an outside observer (although the performer lacks any sense of how results are achieved).

## 4. Epistemic engineering in a working environment

Next, we turn to vicariant effects that arose when drones were introduced to a Danish utility company. Similar to the cyborg-cockroach approach, parts use epistemic engineering within a practical assemblage (Nail, 2017) that can be (partially) described by distributed cognitive systems^7^. The changes both draw on–and favor–vicariance as agents change both how they act and/or what they know. While natural evoneering occurs, in this case, agents often also gain a “grasp” of their place in changing public practices. As shown below, this applies especially to a system operator whose work is pivotal in the working environment. Drawing on the experience of other tasks (i.e., of a pre-drone task regime), he brings forth new possibilities. As a result, human participants grant systems and parts new functionality that, in practice, constitutes valid knowledge. They use an experience-based sense of events, or the feeling of what happens, to actualize practices. Furthermore, they discuss the results and use their talk to adjust later behavior, alter systemic function, and, thus, the use of parts, materials, and a task regime. In this case, there are no new intents. However, just as with the cockroach, the change reduces to neither planning nor the automatization of skills. Rather, it arises from *grasping* how systems can bring forth new kinds of functionality.

### 4.1. Pursuing vicariance in a Danish utility company

In Denmark, district heating supplies most urban environments and is used by 64% of all households. With such heating, hot water is pumped from combined heat and power plants through distributed stations to private homes, businesses, and public institutions. After reaching its destination (i.e., the radiators of the structure to be heated), the “used” water returns for re-heating *via* a network of pipes. While ideally closed, the system suffers from spillage and, for this reason, companies have to add make-up water (and consume extra energy). For this reason, to reduce, or prevent, such leakages without changing pipes, a crucial role falls to the work of the maintenance department. In 2016, the utility company in question began collaborating with a provider of drones that use thermographic cameras for leakage detection. The cameras readily detect the changes in heat radiation from water that is pumped at around 80°C: once the information is identified, heat radiation from underground pipes can be rendered “visible.”

Many different practices^8^ contribute to the maintenance of the pipe network. In this context, therefore, we stress that the introduction of drone technology has cascading consequences. Indeed, the prominence of leakage detection has vicariant effects across the company. To us, it appears that drone-based effects are transforming the mission of maintaining the pipe network. For now, we track innovation in a bundle of practices (i.e., maintaining the pipe network) that, in return, have fed both across other work and back into the use of drone-facilitated information in the maintenance department. In the subtask regime that has arisen, the use of drones (1) creates a novel task (i.e., thermographic leakage detection); and, (2) qualifies an existing one (e.g., the repairing of leakages) relates to the mission of maintaining the pipes. Unplanned changes thus have far-reaching consequences because existing work must both fulfill extant task regimes and, at once, alter in responding to use of drone cases. Hence, drones have become increasingly central to maintenance practice, changed relations between employees and external contractors, and prompted senior management to set a weekly target for dealing with drone cases. The vicariant effects are unplanned because, rather than integrate the drone task regime with extant practices, they have had to be improvised. They have been brought in piecemeal both to supplement general operations (i.e., “non-pipe related maintenance tasks” such as the change of manhole covers) and in changing the pipe network maintenance (e.g., the repairing of alarm threads in certain pipe types). For ease of exposition, we now draw a comparison with the minimal case by identifying the outward flow of vicariant effects.

**Figure 4:**
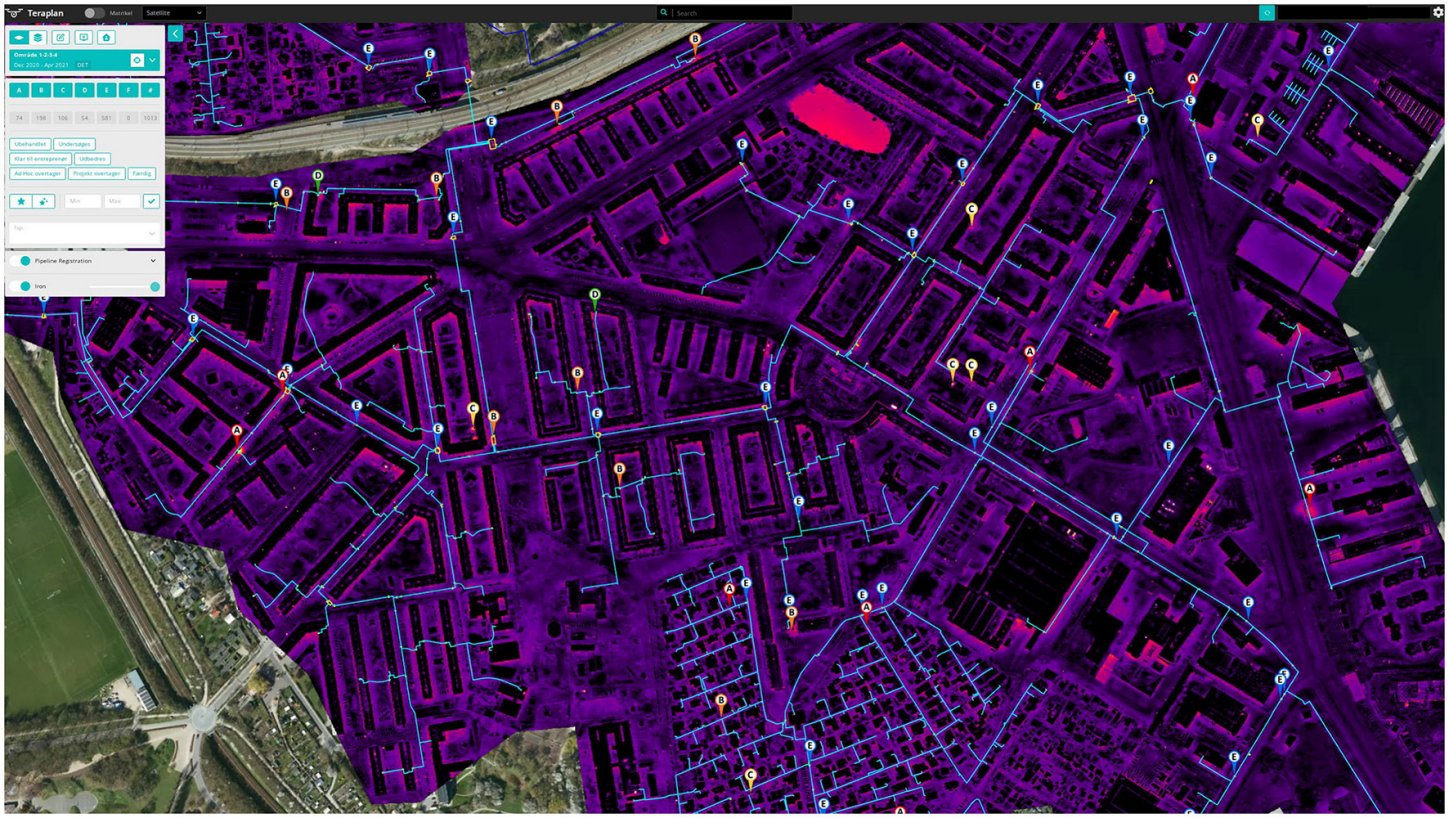
Screenshot of Teraplan.

Over time, seeing the images triggers a cascade of vicariant effects (leading to both intra-organizational change and effects on sub-contractor operations) (see Figure 4). Under the old task regime, decisions about repairing leaks drew largely on contingencies. Since the utility company had no means of seeking out leakages, they relied on when, for instance, a vigilant citizen found green water in their basement (the make-up water has added green color) or if, following a snowfall, an expert noted melted snow above an underground heating source. Hence, drones brought a new order to their work^9^. Furthermore, since they have proved both reliable and efficient, the leakages could have potentially overwhelmed the department's financial, human, and other resources. As one senior manager says: “The drones give us knowledge of leakages that it would otherwise take 10–15 years to gather” (Senior manager). As so often with digital solutions, the accumulation of data demands epistemic engineering and, at once, sets off epistemic change. Having seen that drones bring about new functionality, senior management set the target of addressing 5 new drone cases each week.

### 4.2. Drone task regime: Screening and managing of incoming data

The utility company uses a drone service provider as a semi-autonomous assemblage that provides images based on the specialized software (see Figure 4). Given a technical specification, the parts couple tightly with the company's task regime: employees quickly established the routines based on the classification of suspected leakages. The service provider package includes (a) aerial surveillance of areas of the city and then (b) thermographic images from the surveillance operations supplied to through licensed, custom-built software: *Teraplan*. In the case of (b), the Teraplan data are the drone provider's extension of Google Maps to classify the suspected leakages on a certainty scale (*viz*. As are most certain, Bs less so; and Cs are call for further examination). Furthermore, the user can turn software layers on and off (i.e., to focus on the thermographic layer, Google Maps satellite photographs or the utility company's network of pipes; refer to Gahrn-Andersen, 2020). Plotting of the suspected leakages is performed manually by the drone operator who screens thermographic images while using a depiction of the utility company's network of pipes.

For the maintenance department, Teraplan sets off vicariant effects. Since these must be monitored and managed, the program is shaping an unplanned task regime. In this context, the role of the system operator takes on new importance. Above all, this is because the role now combines extant knowledge and skills (e.g., knowledge of the streets of the city) with a grasp of what Teraplan shows. Drone-based information combines with personal knowledge that draws on the company's own Geographical Information System (GIS). Rather as with the cyborg cockroach, images-cum-software demand that the system officers attune to the output of Teraplan. Bodies function as parts of an adaptor (just as, elsewhere, a Morse operator's body comes to act as an adaptor, see Cowley, 2019). While we later highlight contrasts, parties close to the software are required to develop techniques (not described here) that, oddly, bring new understanding to the old experiences. The resulting decision-making alters the parameters of action and, thus, company practices. We begin with how, given the accuracy of leakage detection, the system operator sets off epistemic engineering. Given his grasp of how drone-based information bears on the wide system, he has to (1) verify the leakage indicated and (2) initiate repairing by forwarding relevant information to the sub-contractor.

Since Teraplan indications of leakages are accurate, the system operators developed a distinctive routine. They link the output to professional knowledge and the utility company's GIS system to set off vicariant effects across the whole system (i.e., the rest of the maintenance department, relevant contractors, the municipality, and private citizens). The resulting epistemic engineering is achieved by acting in ways that favor leakage repair: just as with the cockroach, epistemic change arises as parts of the assemblage exert co-control. These are funneled by how the service provider's coders process raw data and, above all, the system operator's validations and decisions. In what follows, we focus on suspected leakages that are classified as As. While the classification has identified hundreds of successful cases, there are also errors. For example, one A identified ground that had been heated up by a parked bus, and in another case, it showed clamping close to the surface as shown on the utility company's GIS depiction of pipes. Accordingly, the system operator makes an experience-based assessment of each leakage: information from Teraplan is verified by a double check or, as a system operator says: “[The drone] doesn't know what is underground. The GIS [Geographical Information System] does.” While Teraplan can show whether a suspected leakage is close to a pipe, the GIS system adds detailed information about each pipe's type, dimensions, exact lengths, etc. Hence, the system operators compare the Teraplan images with the information from the GIS. They use personal knowledge to identify false positives such as when increased thermographic radiation on clampings does *not* show a leaking pipe. Hence, one system operator, a smith with years of hands-on experience, stresses the need for fine comparisons between images from the two information systems:

As long as we have these two systems [i.e., Teraplan and GIS] like this, it is fairly simple to work with them. Because I also think that we need to keep ourselves from accessing this one [i.e., the GIS] too much. In spite of it, it is a webpage which runs constantly, and our GIS system is so massively huge, you know. It is a way heavier system [than the drone operator's software]

Having double-checked the Teraplan data with the GIS, the system operator also draws on his own experience in deciding when to authorize the utility company's contractors to start on any given case. As confirmation, the contractor begins with a preliminary digging to validate the accuracy of the spot identified. Additional measures require that a system operator or contractor visits each suspected leak and verifies the results using a handheld thermographic camera. However, given the precision of coding As, this procedure has become little more than a formality. Leaving aside work with Bs (let alone Cs), we now turn to how, in the second part of the drone task regime, important contrasts arise with the cockroach case. This is because, as vicariant effects fan out from the system operators, they lose predictability: managing repairs requires entangled links between organizational settings and, thus, care in adapting parts of the assemblage as one manages distributed agency.

### 4.3. A secondary dimension of the assemblage: How the repairs are managed

Whereas opening the drone case has become part of a routine, the subsequent management of repairs is rather loosely structured. Much depends on a weekly “damage meeting” [Da. Havarimøde] where the maintenance work is organized. The meeting enables drone task work while also dealing with both pipe and non-pipe-related maintenance. Each case is given status updates and, where works are not progressing, solutions are brought forward. The logic of each repair is roughly this: (1) the contractor applies to the municipality for permission to dig; (2) affected customers are notified of heating disruption; (3) once a leakage is dug free, its extension is approved by a system operator (who might also chose to temporality close the hole). Later, when the pipe can be replaced by contracted pipe specialists, (4) the utility company sends out a technician to turn of the water. In step (5), the contractor replaces a section of the pipe, and, in (6), the utility company technician restores the flow. Next, in (7), the digging team fills up the hole, lays new asphalt, and removes barriers and signs. Finally, in (8), the utility company technician fills out a “damage report” [Da. Havarirapport] that documents the works and serves to update information in the GIS. In actual circumstances, of course, the progression can be negatively affected by the factors such as staff shortage, an overload of cases, or unforeseen events (e.g., frost that makes digging difficult). In what follows, we present two drone cases reported at a damage meeting held on 26 March 2019:

Drone case 1:

Digging commences in week 49. 11-12-2018: Digging in week 50 because we did not manage in week 49. 18-12-2018: Digging commences 19.12.18. 08-01-19: The digging permit [which is temporary and issued by the municipality] has been reevoked due to expiration. A new hearing phase has started. 15-01-2019: hearing is ongoing 22-01-2019: hearing ongoing. 29-01-2019: Digging permit received, commencing in week 6.05-02-2019: Digging d.6/11. 12-02-2019: Digging. 19-02-2019: Waiting due to parked car. 26-02-2019: Still waiting because of the car. 05-03-2019: Waiting due to parked car. 12-03-2019: digging completes in this week 11. Is being planned. 19-03-2019: digging finished. 26-03-19 status unknown.

Drone case 2:

Ready for [the contractor]. Contact the customer prior to commencing. 29-01-2019: Shooting pipe [a type of pipe] 22.05-02-2019: Expected beginning in week 7. 12-02-2019: [Manager 2] follows up with [digging contractor] in relation to the commencing. 19-02-2019: Commencing Friday 22/2. 26-02-2019: commencing week 9. 05-03-2019: Commencing 06.03.19 12-03-2019: A Greek in place [a term for a temporary repair of the leakage] 12/3. Expected clearance digging week 11. 19-03-2019: [Utility company technician] is to contact [digging contractor] regarding eventual repositioning of the plug. On the agenda for the supervision meeting 22/3. 26-03-2019 digging continues

Notably, the meeting focused on 18 drone cases: as was now clear, the utility company had inadvertently caused a bottleneck. This is because, without having any means of tracking vicariant effects, senior management had introduced a target of five drone cases a week. Given the unplanned nature of the process, additional drone cases were issued to contractors on 12 March and, by the time of the meeting, the bottleneck had been developing for a month. Indeed, for reasons that cannot be discussed here, the continuous addition of new drone cases led to unexpected difficulties for, above all, the digging contractor. Subsequently, the utility company was to react by temporally suspending its “five leakages per week” policy. The two cases serve to illustrate the problems and give a sense of what, precisely, is meant by saying that drones led to epistemic engineering as systems and parts developed functionality that, for those in the company, constitute valid new knowledge.

In the first case, 3 months had passed in progressing from steps 1–2 to the operational repair procedure. This was due to two unforeseen tasks: (a) renewal of the digging permit and (b) the need to remove a parked car which, in fact, led to a 2-month delay before digging could begin (the reason for this was that the company then faced issues with expired digging permits and material and manpower shortage). Whereas, the need to reapply for the permit is a dysfunctional element due to shoddiness and lack of manpower, the second case is a common contingency that, in this case, led to a serious delay. By placing a “Greek” on the pipe, the utility company successfully completed step (3). Yet, since more coordination (i.e., a “supervision meeting”) was needed, an emergency *ad hoc* meeting was called to deal with cases that were piling up because a contractor had fallen far behind schedule. In this particular case, both the contractor and the utility company had overestimated the duration of repairs, and conversely, underestimated how maintenance operations would be influenced by environmental factors.

**Figure 5:**
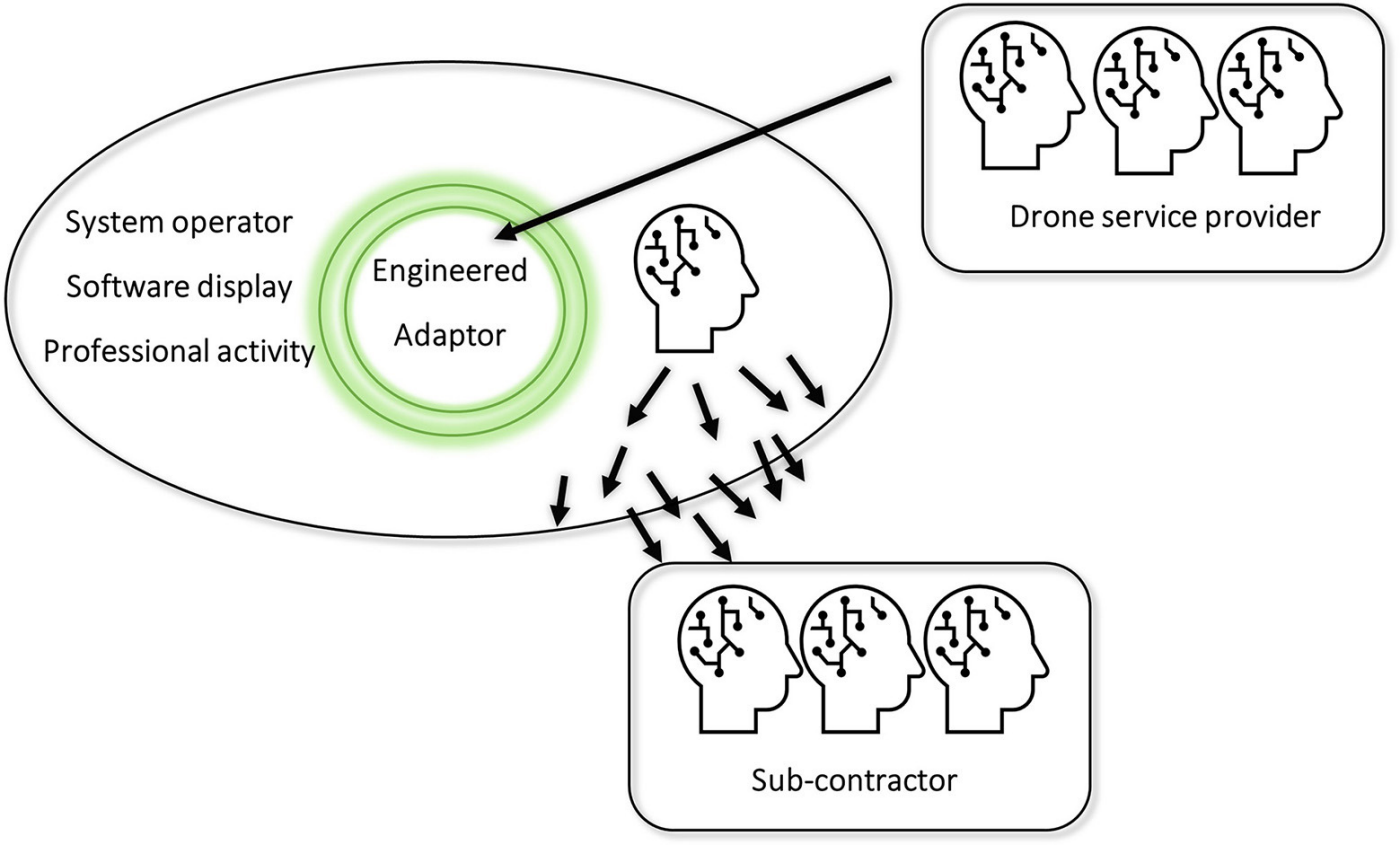
The drone service provider (outer source) enables a system with an engineered adaptor. As a whole adaptor system, the system operator, the software display, and professional activity set off epistemic change.

Unlike the minimal system, the utility company's systems are, at once, organized and deeply entangled. They arise in a poly-centered unit that includes people with very variable understanding. The results have the indeterminacy of *systemic assemblages* (Gahrn-Andersen, 2020) that are: (1) open to social, market, and technological change; (2) enable drones and information to produce functionality; and (3) bind the causal, the biological and social. As we see, drone functionality is fully entangled within organized life: it includes, first, coders (and drone operators) who plot useful data in Teraplan; second, it has made the system operator who uses the software into an “adaptor” like a person with a verifying/facilitating role. However, the assemblage must cope with not only drone-derived data but also seemingly drone-independent repercussions that are conceptualized around the tasks of repair. Indeed, given poly-centered control, as in similar organizations, the utility company uses a hierarchical structure to maintain institutional control (e.g., through damage meetings). In clarifying how parties manage epistemic engineering, therefore, we draw contrasts to the minimal system. Whereas, the human-cockroach adaptor is encapsulated, Teraplan makes the system operator into an adaptor whose functionality disseminates. To an extent, diverse, loosely coupled systems demand from the other human parts that they adjust their ways of acting (and develop novel techniques). Above all, skillful agents (the drone operator's coders and the utility company's system operators) determine the company's function and operation. Hence, in moving from a drone-specific task regime to the maintenance task, the task coupling becomes looser and, at times, decouples (at least in part). In such cases, additional supervision meetings are needed (cf. Drone case 2). In bringing order to such events, we now consider the implications of recognizing how adaptors set off vicariant effects. We stress that, since epistemic change is incorporated into action, talk, and routines, human cognition can use how intents and epistemic change arise in socially organized wide systems (refer to Figure 5).

## 5. Organized humans: A systemic view

Complex systems such as toy locomotives and galaxies contrast with the bodies that subserve human knowing. As Bateson (1979) notes, “the toy locomotive may become a part in the mental system which includes the child that plays with it, and the galaxy may become part of the mental system which includes the astronomer and the telescope (1979, p. 104).” In his terms, objects are not thinking subsystems in larger minds but, rather, nature evolves as observers (or knowers) use *relationshi*ps. Overlooking entropy reduction, he suggests that these arise “between two parts or between a part at time 1 and the same part at time 2 (p. 106)” and activate a third component such as a sensory end organ. The receiver “responds to is a *difference* or a change” (Bateson, 1979). Receipt of the differences makes a difference for a system. In parallel, for Giere (2011), there is an asymmetry of knowing and cognizing. As illustrated by the Hubble telescope, whereas cognitive outputs (e.g., images from space) derive from the whole system, only human parts can *know* anything. This asymmetry is fundamental because of the clear implication that bodies use cognitive input to create an epistemic output (differences that makes a difference for a system and/or its parts). In Bateson's terms, distributed systems use “differences” or information that the doings of living parts transform into knowledge and know-how (as things happen). Yet, Giere leaves aside *how* “receiving” can prompt coming to know. In addressing this in humans, we suggest that knowledge arises in wide systems as living parts reduce entropy, simplexify (Cowley and Gahrn-Andersen, 2022) and make use of adaptor systems.

As epistemic actors, humans both receive and process information (or perceive differences) as they exert control over the results. In focusing on how cognition binds human understanding with the deliverances of wide systems, we take a systemic view (Cowley and Vallée-Tourangeau, 2013, 2017). As with the cockroach controller or the drone system operator, epistemic change uses systemic interdependency. Whereas, cognizing pertains to a whole system, *knowing* concerns Giere's (2004) “human cognitive agent” or, simply, a living human being. The move resolves the collective-individual tension noted by Baber (2010), Perry (2013), and Jones (2013) by making artifacts and language part of a distributed agency. As shown by Fioratou and Cowley (2009), for example, insight problems are solved as bodies are nudged to abstract “aspects” from lived experience. In Cowley and Vallée-Tourangeau's (2017) terms, primates “notice things” by drawing on what is called the principle of cognitive separability (PCS). In noticing, we take distance from body-world engagement as doings attune to *aspects* of things. In tool use, for example, we “try” things out and, with experience, learn from practice (Donald, 1991). Given distancing (and the PCS), a contingency can prompt *seeing* a solution (Ball and Litchfield, 2017) or problem-solving can be triggered by the aesthetics of symmetry (Steffensen et al., 2016). Positing the PCS both clarifies epistemic outcomes and also shows the cognitive value of attending to emplaced experience. Together with distancing, one can generate intent and epistemic change using interactivity (Kirsh, 1997; Gahrn-Andersen, 2019), resonating with pico-dynamics (Blair and Cowley, 2003) or striving for cognitive events (Steffensen, 2013). The PCS links routine performance with higher cognitive functions (Cowley and Vallée-Tourangeau, 2017). Yet, appeal to a principle leaves aside how living parts of wider systems change parameters with epistemic effect. After all, only *some* events shape techniques and only expertise can derive useful outcomes from systemic interdependencies. It follows that distributed systems do not just self-sustain but, just as importantly, co-function as persons, brains, and bodies generate epistemic change. Given distancing, attention, and emplacement, people draw on a life history to exhibit powers associated with what Madsen (2017) calls multi-scalar temporal cognition. In a Mafia setting, for example, a mother may desecrate her child's “informer's grave” (Neumann and Cowley, 2016). Coming to “know” the appropriacy of such action eludes both neurophysiological or convention-based accounts (i.e., micro- or macro-explanation). Rather, the desecration attests to an organized domain where human agents link the micro with the macro. As a member of the Mafia world, the mother is concerned with neither a task nor a distributed cognitive system. Damaging her child's grave is inexplicable by accounts based on either interaction history or normative social roles. Rather, events presuppose a public space of action where wider systems operate as constraints on neurophysiology and, thus, action: adjustments unite public appearances (and responding to them) with the macro-social and the bio-behavioral. Formally, one can posit the three co-functioning dimensions (Secchi and Cowley, 2016, 2021; Secchi et al., 2023) known as the Ms (macro, micro, and meso). In peer review, for example, a reviewer drives epistemic change by drawing on organized structures, individual prompts, and judgments of what is likely to be perceived as having scientific value (Secchi and Cowley, 2018). Tasks and cognitive ecosystems become part of a meso-domain–a public space of unending, structural change.

A focus on structural change privileges systemic interdependency. As in the Mafia case, behavior is irreducible to interaction. People simplexify or reduce entropy by drawing on retroactive processes. They amalgamate past experiences with a lived now both in willing cockroach movement (using techniques) and binding Teraplan images with “knowing” the streets shown by the GIS software. Within a meso-domain, one acts as *a person in the system* (Fester-Seeger, 2021). As parts of wide and distributed systems, in Bateson's (1979) terms, people recognize the differences and enact news. As Hutchins sees, they reduce entropy and, we add, set off vicariant effects that *make* differences. The claim matters in that it addresses Afeltowicz and Wachowski's (2015) objection to the distributed perspective. Intents can be public, multiscalar effects that embody epistemic changes. In the cockroach experiment, an engineered adaptor prompts an experimental subject to develop purposeful behavior. While brain-enabled, *contra* Afeltowicz and Wachowski (2015), thoughts need, not a neural mechanism, but a special *way* of “looking while willing.” The brain creates novel structures (techniques) within a wide system where a person becomes part of an adaptor system that controls the brain-cockroach whole. In the utility company, a system operator achieves epistemic outcomes by retrojecting the experience of terrain onto a software display. As an expert, he can see that Teraplan shows a bus stop that is “too far” from the side of the road. In such a case, expertise can prompt one to challenge evidence. Cognizing thus arises in the meso-domain of an extended system: this is where the experimental subject makes the cockroach turn and the system operator decides to check an intuition at the site specified. While brain-enabled, the action is reliant on public cues; the brain's role is, not to control, but to grant a sense of purpose (i.e., as in a Tolman agent). In the wide system, the cockroach controller amalgamates changing impressions (the system in the person) with increasingly effective action (independent of belief). In parallel, organized routine co-functions with equipment to form a system operator's intuition. Furthermore, while the PCS plays no role in the action, the techniques presuppose an enlanguaged world (refer to Cowley and Gahrn-Andersen, 2022) where actions make sense: this enables a person in the system to see what can be done or grasp what one is meant to do.

Sensitivity to the moment is the hallmark of social organizing. It allows the persons to attribute a public (or “relevant”) sense to events and, thus, establish vicariant effects. Hence, living systems use systemic interdependencies to shape the “outward spread” of knowing. In the drone case, the spread affects a range of stakeholders as persons reduce entropy through epistemic engineering. While using routines and cultural ecosystems, parties also develop techniques and act to simplexify. Without knowing what they are doing (or explicit training), they alter both whole system functions and also those of bodies and living persons. Epistemic change can reveal what one “should” do or prompt a grasp of the possible. Often, experience, expertise, and techniques bind with what linguists call entrenchment (Cowley, 2017; Schmid, 2020). The resulting judgments use, not a faculty of reason, but how practical know-how unfolds in an enlanguaged world. Experienced individuals gain capacities for reliable judgments and making use of docility (Secchi, 2016). In the utility company, these qualities–not just routine use of systems–ensured a smooth transition to drone use in pipe maintenance. A well-organized systemic whole ensures that drone-based information is currently driving the reorganization of maintenance work (Gahrn-Andersen, 2020). As change spreads, people link bodily feel and expertise to causal systems that set off cumulative practical effects. The equipment serves, not just directly, but also to improvise new material and institutional relations (i.e., by setting boundary conditions on sensitivity to linguistic semiotic resources). The vicariant effects enable the teams and individuals to (a) self-empower; (b) reorganize; (c) influence each other; and (d) alter routines. Parties gain expertise, skills, and ways of drawing on the system. Thus, while many new issues arise (e.g., reorganizing supply and budgeting needs), the drone study also shows how resilient organizations and individuals gain from cascades of epistemic change.

## 6. Epistemic engineering

Emphasis on systemic interdependencies plays down the role of organism-centered control. Indeed, the radical potential of the systemic view lies in bringing a constructive role to distributed systems. As we have argued, they enable humans to generate intents, epistemic effects, and collective knowing: often persons *make* differences using wide systems to set off vicariant effects. During routines or practices we enact and mimic adaptor systems that trigger epistemic change. Hence, agency and tasks are reciprocally related. The view clarifies how wide systems contribute to social intelligence in lemurs (Jolly, 1966; Sterelny, 2007), navigating a ship (Hutchins, 1996), or using “thought control” over a cyborg cockroach. In hominins, neural plasticity co-evolved with new variation in cognitive performances: at times, we attend closely and, at others, we distance ourselves and, given hints, gain insights (“perhaps a bus warmed the ground”). In part, this is due, we suggest, to the principle of cognitive separability that allows us to notice potential value in the contingent. Indeed, without it, there would be no flexible-adaptive tool use or amalgamation of social regularities and irregularities. By implication, the epistemic novelty of hominins may derive from our use of distributed agency. With the rise of artifice, humans come to draw on, not just bodies, but also reciprocal relations within wide systems and across practices.

In an enlanguaged world, vicariant effects contribute to intents, routines, and practices. In the “minimal” case, a person-in-the-system sets off epistemic change by purposefully moving a cockroach. In the system, looking-and-willing reduces entropy as a brain adapts to the engineered adaptor. In the utility company, epistemic change reaches beyond techniques as drones cum Teraplan software enable a system operator to set off a cascade of effects. In this case, epistemic engineering prompts people to see opportunities and, over time, figure out what to do: while requiring neural re-use and control, the power of self-sustaining systems (and the meso-domain) lies in generating useful knowledge. By enabling adaptor systems, we use epistemic effects to get things right. Without any foresight, people link entropy and the pre-reflective with the hints and nudges of an enlanguaged world. Within interdependent and distributed systems, vicariant effects enable epistemic change, self-empowerment, new uses of equipment, co-creativity, and variation in routines. We, therefore, submit that much is gained from teasing apart living agency from that pertaining to supra-systems, tasks, and routines. The radical move allows cognitive powers to use, not only bodies, brains, and organism-environment coupling, but how human life cycles serve in making differences. The biosocial resources of wide systems can be used to ensure that distributed control sets off vicariant effects whose parameters function to *construct* epistemic change. In short, while selection filters novelty, non-linear change transforms the knowable. By hypothesis, then, epistemic engineering is an evolutionary principle that may well apply across the living world.

## Data availability statement

The original contributions presented in the study are included in the article/supplementary material, further inquiries can be directed to the corresponding author.

## Ethics statement

Ethical review and approval was not required for the study on human participants in accordance with the local legislation and institutional requirements. The patients/participants provided their written informed consent to participate in this study.

## Author contributions

Both authors listed have made a substantial, direct, and intellectual contribution to the work and approved it for publication.

## Footnotes

^1^The system measures “steady state visual evoked potential” as EEG response from the visual cortex that arises in looking at the moving cockroach on a flickering screen. The EEG system is adjusted to focus on a certain bandwidth. Hence, what we call “looking and willing” involves a range of factors and, as with any such system, there are issues of noise. Thus, while subjects are asked to keep their heads still, even in the demonstration video, they track the cockroach movement in ways that are highly visible.

^2^The develop a *control performance coefficient* to contrast system performance as compared to chance (or a control). They tested the mean CPC value of 0.616 ± 0.169 against the chance level (0.375) with a one tailed t test and found it was highly significant, citing a t *p* < 0.0001 (t = 8.170).

^3^Given longitudinal data, we could not track the role of “watching and willing” or how “noise” affects classification of measures. We do not attempt that here.

^4^Gahrn-Andersen and Prinz (2021) suggest that, for the human part of the system, the brain's enabling activity is part of the “pre-reflective.” Since one feels about what one sees one needs no “representations”. This is possible, they suggest, because hierarchies of molecular coding draw on (and, perhaps re-use) configurations of electromagnetic and cognitive patterns. The brain may combine the use of more meaningful peripheral elements with a computational core.

^5^In Dennettian terms, Skinnerian agents link a history of reinforcement together with planning and selection such that, in some species, culturally transmitted replicators sustain “off-line” learning. Unlike Popperian agents, they lack “models” of the world: in developing an *ex novo* technique, one needs neither cultural replicators (instructions) nor a model that corresponds to an external environment. Presumably, the novel technique arises from a (coded) reconfiguring of neural sub-systems (or what Piaget calls accommodation) as well as reinforcement. Importantly, one need not know that one is controlling EEG input; change happens *for* a person (who can falsely believe they rely on “thought control”).

^6^The label is in Dennettian spirit. While alluding to Tolman (1932), we do not suggest that such agents act in accordance with his theory. Simply, they use the law of effect to act in ways that, seen from an intentional stance, appear purposeful.

^7^*Assemblage* is used in translations of Deleuze and Guattari who apply the term to characterize parts that co-function neither in ways predetermined to fit an already-conceived design nor a random collection of things see, Nail (2017). Where parts align with functions they can be described as a distributed cognitive system.

^8^Indeed, the utility company's history of proving district heating goes back to 1925.

^9^Here, we are looking beyond leakages that are automatically reported by the alarm threads in certain pipe types. The drones have been introduced with the purpose of spotting leakages in pipes that do not come equipped with such threads.
